# Gonadotropin-Releasing Hormone Antagonists—A New Hope in Endometriosis Treatment?

**DOI:** 10.3390/jcm12031008

**Published:** 2023-01-28

**Authors:** Anna Maria Rzewuska, Monika Żybowska, Ilona Sajkiewicz, Izabela Spiechowicz, Klaudia Żak, Monika Abramiuk, Krzysztof Kułak, Rafał Tarkowski

**Affiliations:** 1 I Chair and Department of Oncological Gynaecology and Gynaecology, Student Scientific Association, Medical University of Lublin, 20-081 Lublin, Poland; 2 Independent Laboratory of Minimally Invasive Gynecology and Gynecological Endocrinology, Department of Oncological Gynaecology and Gynaecology, Medical University of Lublin, 20-081 Lublin, Poland; 3 I Chair and Department of Oncological Gynaecology and Gynaecology, Medical University of Lublin, 20-081 Lublin, Poland

**Keywords:** endometriosis, GnRH antagonists, medical treatment for endometriosis, pelvic pain, dysmenorrhea

## Abstract

Endometriosis is a chronic disease, in which endometrial-like tissue is found outside the uterine cavity. Lesions are typically located in the true pelvis but can be found, in addition to extragenital endometriosis, in the respiratory system, the diaphragm, the pleura or the pericardium. As the extrauterine endometrial lesions undergo the menstrual cycle, they cause many symptoms, including pain, and besides infertility, they all mostly affect the quality of the patient’s life. Pharmacological management of endometriosis significantly increases in importance either as a first-line treatment or as a complementary therapy after surgery. Yet, current research on antagonists of the gonadotropin-releasing hormone (GnRH) has revealed their potential benefits in endometriosis treatment. Their mechanism of action is to down-regulate the hypothalamic–pituitary–gonadal axis and therefore induce a hypoestrogenic state. The resulting reduction of estrogen levels prevents disease progression and diminishes the recurrence rate after surgical removal of endometriosis. The present review summarizes recent reports of the role oral GnRH antagonists have as a significant treatment option for pain reduction in endometriosis patients.

## 1. Introduction

Endometriosis is a chronic condition in which endometrial-type mucosa (specifically stromal and glandular cells) is found outside the uterine cavity and undergoes exfoliation during menstruation. For now, despite the high number of patients with endometriosis, the pathogenesis of this disease is not completely understood. Several hypotheses have attempted to explain the development of this condition. The most popular theory is based on retrograde menstruation. It claims that the menstrual endometrial tissue migrates to the abdominal cavity, where it can implant and proliferate [1,2,3]. The second theory, the theory of coelomic metaplasia, indicates that the normal peritoneal tissue may transform into the ectopic endometrial tissue when it is hormonally stimulated [3]. Importantly, endometriosis can also be found outside the uterine cavity in locations such as abdominal lymph nodes, lungs, pleura and others. This process is explained by the metastatic theory, which indicates that menstrual tissue may be transported through lymphatic channels and veins from the endometrial cavity to distant sites [2,4]. Moreover, the endometriotic tissue demonstrates both an increased activity of estrogen receptors and decreased resistance to progesterone, which consequently leads to the impairment of immune function, an increase in inflammatory rate and the impairment of apoptosis [3,5,6]. About 10–15% of women of reproductive age suffer from endometriosis [7]. Many patients remain undiagnosed for many years—the average time between first symptoms and confirmed diagnosis is 4–11 years [8]. Most commonly, endometriosis is diagnosed in women between the ages of 25 and 45 years old [9], and the average age of diagnosis is 28 years old [10]. However, there are also cases of patients with endometriosis in the menopausal period and at the age of puberty [11,12]. The literature also reports the occurrence of foci of endometriosis in fetuses [13] and isolated cases of endometriosis in men who have undergone hormone therapy for prostate cancer [14].

The localization of endometriotic lesions can vary, and it can manifest itself with numerous symptoms, such as dysmenorrhea, hypermenorrhea, gradually increasing acute premenstrual pain, pelvic pain, dyspareunia, painful ovulation, pain while defecating and/or when urinating, nausea and abdominal bloating; therefore, the diagnosis is often difficult to make. However, one of the most common symptoms of endometriosis is pain—both associated with the menstrual cycle and independent from the menstrual cycle.

Overall, pelvic pain and dysmenorrhea represent the type of pain typically associated with endometriosis. Dyspareunia is a condition that may affect 30% to 50% of patients and, together with infertility, it has a tremendous impact on sexual life and reproductive plans. The urge to urinate and dysuria are both related to the location of the urinary bladder, while dyschezia, diarrhea and constipation can easily be mistaken for Irritable Bowel Syndrome (IBS). Endometriosis-related symptoms affect productivity at work, everyday activities and social relationships. Thus, clearly, endometriosis negatively affects the physical and mental well-being of women, their quality of life and consequently has psychological effects that can even lead to depression [15].

Diagnosis of endometriosis poses challenges for doctors, and the lack of appropriate diagnostic tools enabling correct diagnosis in medical practice has always been the most problematic. Magnetic resonance imaging (MRI) and ultrasound scans helped diagnose endometriosis but the process was still complicated. In many cases, laparoscopy was the only form of examination that allowed the diagnosis of the disease [16,17]. However, in February 2022, the European Society of Human Reproduction and Embryology (ESHRE) published new guidelines on the diagnosis, treatment and management of endometriosis, where laparoscopy is only recommended in patients with negative imaging results (ultrasound or MRI) and where empirical treatment was not successful [18]. The removal of endometriosis foci is not the same as the elimination of the disease since it constitutes a recurrent and chronic problem. Therefore, the introduction of pharmacological treatment is essential and it should aim to reduce and eliminate pain. This treatment should further inhibit the development of the disease, regression of endometrial foci and finally, it should restore fertility [19]. The first-line treatment options are nonsteroidal anti-inflammatory drugs (NSAIDs) and low-dose combined oral contraceptive pills (COCP) or progestins (oral, injectable and intra-uterine), whereas the second-line treatment includes gonadotropin-releasing hormone (GnRH) agonists and GnRH antagonists. Aromatase inhibitors should be considered as the second/third therapeutic option. According to ESHRE guidelines, the second/third line of treatment is concerned only when the first group of drugs is ineffective or not tolerated. [18]. The mechanism of action of the following drugs is shown in Table 1 [18,20].

NSAIDs are the most commonly used drugs which reduce and control pain, but they do not eliminate the cause of the disease. Hormonal therapies rely on the suppression of the endometriotic tissues, but they have undesirable side effects secondary to hormonal suppression, and such therapies need to be closely monitored. The newest medications, which are currently evaluated in endometriosis therapy, preliminarily with satisfactory results, are antagonists of the gonadotropin-releasing hormone (GnRH) [19].

## 2. Materials and Methods

The studies and research cited in the presented review were selected from PUBMED, Google Scholar and Science Direct databases. Additionally, the websites including https://clinicaltrials.gov/ and https://www.cochranelibrary.com/central/about-central (accessed on 17 December 2022) were checked in detail. The detailed search method is described in the Supplementary Materials (Table S1) added to this article. We excluded articles not written in English, conference abstracts only, clinical study reports, clinical case reports and duplicated papers. We considered reviews and original paper works. No limitations of the year of published study were used and we included literature published until September 2022.

## 3. Scales Used in Order to Measure Pelvic Pain

In order to measure patients’ pain, different scales were used: the Biberoglu and Behrman Score (B&B), the Visual Analog Scale (VAS), the Numerical Rating Scale (NRS), The Composite Pelvic Signs and Symptoms Score (CPSSS), Endometriosis Health Profile-30 (EHP-30), The Endometriosis Health Profile-5 (EHP-5) and Patient Global Impression of Change (PGIC). The use of those scales depends on the geographic region—in Europe, VAS is used more frequently but the FDA recommends the B&B Score and its components [20]. The VAS consists of a 10 cm long horizontal line, in which 0 mm on the extreme left means ‘no pain’, and 100 mm on the extreme right means ‘worst pain imaginable’. The NRS is quite similar to the VAS, but it uses numbers from 0 to 10 to measure pain. The B&B Score asks about three symptoms: dysmenorrhea, dyspareunia and chronic pelvic pain, and two signs: pelvic tenderness and induration. Each of the above-mentioned may have from 0 to 3 (or 4) points. Higher numbers indicate a more severe pain experienced by women. The CPSSS is a modified version of the Biberoglu and Behrman Scale [21] and assesses dyspareunia, dysmenorrhea, non-menstrual pelvic pain (NMPP), pelvic tenderness and pelvic induration. The total CPSSS is calculated as the sum of the total component scores. The EHP-30 is the first standardized instrument evaluating health-related quality of life in women with endometriosis [22] and contains a 30-item questionnaire and five scales: pain, feeling of control and powerlessness, emotional well-being, social support and self-image [23]. The EHP-5 is a shorter version of the EHP-30 [24] and relies on a self-report questionnaire in which patients evaluate their quality of life with endometriosis, referring to the four previous weeks. The first part of the questionnaire consists of five items about pain, control and powerlessness, emotions, social support and self-image. The second part contains six items about work life, relation with children, sexual intercourse, medical profession, treatment and infertility. Items are scored from 0 (never) to 4 (always). Total score is then transformed on a scale with a range from 0 to 100 [25,26,27]. Overall improvement during endometriosis treatment can also be assessed by using PGIC on a seven-point scale (1 = very much improved, 2 = much improved, 3 = minimally improved, 4 = not changed, 5 = minimally worse, 6 = much worse, 7 = very much worse) [28].

## 4. Mechanism of Action of GnRH Analogs

GnRH is the superior hormone which controls mammalian reproductive physiology. Three types of GnRH have been described: GnRH type I (GnRH-I), GnRH type II (GnRH-II) and GnRH type III (GnRH-III), which are different in amino acids collection [29]. GnRH is immunoreactive in all endometrial cell types [30], in the epithelium of the fallopian tube [31], in granulosa-luteal cells, the ovarian surface epithelium (OSE) [32], in ovarian carcinoma cells [33], in the placenta [34,35] and in preimplantation embryos and the mass of blastocyst [36]. GnRH may also be overexpressed in breast cancer; therefore, the GnRH-based cancer treatment has been introduced [37,38]. For now, GnRH analogs are used in different gynecological indications, including uterine leiomyomas [39], in in vitro fertilization (IVF) and embryo transfer [40], central precocious puberty [41] and in the treatment for reproductive tract carcinomas [42].

**Figure 1:**
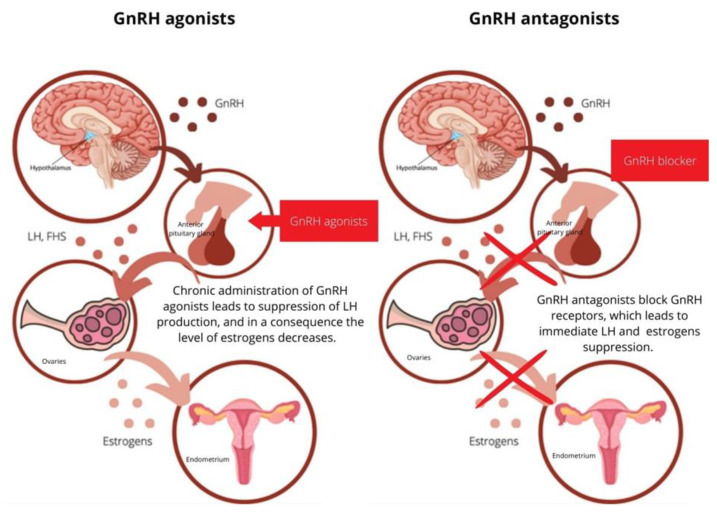
Mechanism of action of GnRH analogs. Prolonged activation of GnRH receptors by GnRH agonists leads to gonadotropin secretion suppression. As a consequence, the secretion of estrogen is also decreased. Comparatively, the mechanism of GnRH antagonists is competitive in the occupancy of the GnRH-receptor. As a result of the blockade of GnRH receptors, the suppression of gonadotropin and estrogen is observed.

The introduction of GnRH-I analogs of molecular structure modifications, including agonists and antagonists, was synthesized. The action of GnRH agonists depends on the reversible blockade of pituitary gonadotropin secretion. The proper action of this treatment requires 7 days or more of constant exposure; therefore, its use is more difficult compared to GnRH antagonists which immediately suppress gonadotropin secretion. Seemingly, GnRH antagonists are as successful as GnRH agonists and also include such advantages as shorter treatment time, lower risk of ovarian hyperstimulation syndrome, less consumption of FSH and improved patient acceptance [29,43,44]. The mechanism of GnRH agonists and antagonists is present in Figure 1.

GnRH antagonists work by competitive action on GnRH receptors, which are present in endometrial cells. Compared to endometrial cells of women without endometriosis, eutopic endometrial cells of women with endometriosis are marked by an increased proliferation rate and lower susceptibility to apoptosis. Therefore, the question of how they can impact endometriosis was raised. GnRH antagonists can inhibit the growth and proliferation of endometrial cells by modulating angiogenic and apoptotic mechanisms. It is based on an increase in the expression of the pro-apoptotic cell proliferation, including proteins Bax and FasL, and a decrease in the expression of the anti-apoptotic protein Bcl-2 [33]. Moreover, in patients with endometriosis, the levels of vascular endothelial growth factors (VEGFs) were observed to be elevated both in the peritoneal fluid and in the endometrial tissue [45,46]. The study of Meresman et al. analyzed the effect of GnRH agonist leuprolide acetate (LA) on the in vitro eutopic endometrial cell apoptosis and the release of interleukin-1beta (IL-1beta) and VEGF. The results of their study showed that LA in the dose of 100 ng/mL enhanced apoptosis in endometrial cell cultures and downregulated IL-1beta and VEGF release both in the patients with endometriosis and in the patients from the control group [47]. In vitro GnRH antagonists may be crucial in endometriosis therapy as they change the metabolism of endometrial cells. To conclude, the present review provides an analysis of the GnRH antagonists and their effects on endometriosis symptoms in women, such as pain and worsened quality of life.

GnRH agonists, due to their hypoestrogenic qualities, prove effective in the treatment of endometriosis-associated pelvic pain. However, apart from the therapeutic effects on endometrial lesions, such intervention causes many side effects, commonly including vasomotor symptoms, vaginal dryness and headaches. One of the most severe side effects of long-term treatment with GnRH agonists is osteopenia (loss in bone mineral density (BMD) [48].

To alleviate this undesirable effect, add-back therapy (ABT) is successfully used [49]. This is based on the re-administration of synthetic or natural steroid gonadal hormones, in which circulating levels have been lowered because of pituitary gland suppression. The most commonly used protocols include the administration of progestagen or progestagen with estrogen.

The main purpose of ABT is to achieve the perfect estradiol range, which would both protect bone mineral density and simultaneously eliminate endometrial lesions [48]. Some studies of ABT applied with GnRH antagonists treatment also indicate its efficiency [50].

## 5. GnRH Antagonists in Endometriosis Treatment

### 5.1. Elagolix

Elagolix is a novel, non-peptide, oral, short-acting competitive GnRH antagonist approved by the United States Federal Drug Administration (USFDA) in 2018 [51] for the management of moderate to severe pain associated with endometriosis. The drug is currently also approved for the treatment of heavy menstrual bleeding among patients with uterine fibroids [52]. According to the USFDA recommendation, the duration of treatment for patients with endometriosis depends on the dosage of Elagolix—for Elagolix in the dose of 150 mg taken once daily it should be no longer than 2 years, or for Elagolix in the dose of 200 mg taken twice a–day—6 months [51].

For the first time, the Elagolix efficiency was evaluated in a multicenter, double-blind, phase 2 Daisy PETAL trial. The group of 137 women aged 18 to 49 with endometriosis was divided into the research group taking Elagolix 150 mg daily and into the control group with placebo intake. The results were as follows: in the research group, a greater number of patients reported at least a 30% reduction in pain scores from baseline to week 8 for dysmenorrhea, for NMPP measured using CPSSS and for dyspareunia.

Moreover, during the phase 2 Daisy PETAL trial, women taking Elagolix had significantly lower PGIC scores compared to placebo recipients. At week 8, 60.3% of women, receiving Elagolix reported being “much improved” or “very much improved” compared with 30.2% of placebo recipients. Quality-of-life measures were also assessed in this research. During the 8-week period, Elagolix recipients demonstrated greater reduction from baseline compared with placebo for four of five EHP-5 dimensions: pain, self-image, control and powerlessness, social support. Eventually, both groups of patients who received Elagolix, in open-label therapy, reported similar improvements in all the EHP-5 dimensions for women from Elagolix groups in the double-blind period. Adverse events (AEs) occurred in 9.9% of Elagolix recipients, and the most frequent were nausea, headache and hot flashes. There were no significant shifts observed in lipid profile or BMD [53].

The effectiveness and safety of two different doses of Elagolix for treating endometriosis-associated pain (EAP) were studied in the randomized, double-blind, multicenter, phase 2 Lilac PETAL trial. The number of 155 women with endometriosis were randomized to the three groups: placebo groups, the group receiving Elagolix in the dose of 150 mg or the group receiving Elagolix in the dose of 250 mg daily for 12 weeks. After this period, placebo recipients were rerandomized to Elagolix groups and Elagolix patients continued their trial for 12 additional weeks. There were observed reductions in the monthly mean in NRS in all three treatment groups at weeks 4, 8 and 12. The decreases were greater among Elagolix patients compared to placebo recipients at week 12, but it was not statistically significant. There were also significant decreases in dysmenorrhea measured by a modified B&B scale for 150 mg and 250 mg Elagolix recipients compared to the placebo group during 12 weeks. The reductions in monthly mean dysmenorrhea and NMPP scores continued through weeks 13 to 24. Mean reductions were significantly greater for Elagolix 150 mg compared with the placebo at weeks 8 and 12 and for Elagolix 250 mg compared with the placebo at weeks 4 and 8. Dyspareunia scores lowered between 13 to 24 weeks similar to Elagolix treatment groups during weeks 1 to 12. The EHP-5 was used to assess the quality of life during Elagolix trials, and in all three treatment groups, the improvement in all EHP-5 dimensions was observed. The most significant improvements were reported in the Elagolix 150 mg group [54].

The next study, the randomized, double-blind, multicenter, phase 2 Tulip PETAL trial, also evaluated the impact of two different doses of Elagolix: 150 and 250 mg per day on pelvic pain. The 174 patients with a laparoscopic diagnosis of endometriosis were divided into four groups administering different substances over the period of 12 weeks: two groups taking Elagolix, the third group receiving LA and the placebo group. Patients who earlier received Leuprorelin or the placebo were rerandomized to Elagolix at week 12. There was a reduction in the mean percentage of days with analgesic use among all patients participating in the trial, but unfortunately, the results were not statistically significant. During week 12, all four trial groups reported decreases from baseline in NRS in the case off EAP. Dysmenorrhea was also reduced in 150 and 250 mg Elagolix groups and LA recipients compared with patients receiving the placebo. Unfortunately, changes in NMPP and dyspareunia in the Elagolix treatment groups were not statistically significant, although in the LA group, they were. There were also improvements in all dimensions of the EHP-5 in each patient group. The Elagolix and the LA recipients had larger decreases compared with the placebo. The comparison between 150 mg and 250 mg doses of Elagolix versus LA was statistically relevant and stated a higher LA influence on the pain component of EHP-5 [55].

In the Elaris Endometriosis I (EM-I) and the Elaris Endometriosis II (EM-II), two similar, double-blind, randomized, six-month phase 3 trials were compared: two doses of Elagolix—150 mg once daily (lower dose group) and 200 mg twice daily (higher dose group) with placebo. The trial was conducted among 875 premenopausal women in EM-I and 788 in EM-II. After 6 months of treatment, about 42.1–43.4% women taking the lower dose and 75.3% taking the higher dose had a significant reduction in dysmenorrhea, versus 23.1% of placebo recipients. NMPP was observed by about 45.7% of patients receiving lower doses and by 62.1% receiving higher doses versus 34.9% of placebo recipients. Moreover, women who received 200 mg of Elagolix resulted with a lower use of analgesics at 3 months and 6 months, and they needed rescue opioids less often than those receiving the placebo. Significantly more women taking either dose of Elagolix reported “much” or “very much” improvement on the PGIC scale at 6 months when compared with those taking the placebo. What is more, due to the EHP-30, women from Elagolix groups during treatment experienced better quality of life compared with the placebo recipients, based on the mean change from baseline to 3 months and 6 months [56].

During EM-I and EM-II, at least one adverse event (AE) was reported, and the most frequently demonstrated were hot flashes, headaches and nausea. Patients who took lower or higher doses of Elagolix more frequently reported hot flashes compared with placebo recipients, but their maximum severity was mild or moderate. Elagolix treatment was associated with increases in lipid profile: total cholesterol, low-density lipoprotein (LDL) cholesterol, high-density lipoprotein (HDL) cholesterol and triglycerides. What is more, Elagolix, as a GnRH analog, may also reduce BDM through its hypoestrogenic mechanism [54,57]. During EM-I, about 3.8% of women in the lower dose group and 20.9% in the higher dose group reported decreases of more than 5% in BDM at the lumbar spine, compared with 1.8% in the placebo group. in EM-II, the percentages were 2.3% among 150 mg Elagolix recipients and 16.4% among 250mg Elagolix recipients, compared with 1.1% patients who took the placebo. Although, the long-term risk of fracture was minimal [58].

The Elaris Endometriosis III (EM-III) and the Elaris Endometriosis IV (EM-IV) were extended Elaris Trials. Studies were also double-blind, placebo-controlled phase 3 trials. The aim of Em-III and Em-IV was to evaluate the results after an additional 6 months of treatment. Women who were in Em-I and Em-II on an active treatment used the same Elagolix doses in the extension research, whereas women who were placebo recipients in Em-I and Em-II were rerandomized to each Elagolix group [58]. After one year of treatment, there was a reduction in dysmenorrhea by about 50.8–52.1% in the lower dose group and 75.9–78.1% in the higher dose group. NMPP was decreased by about 66.4–67.8% among 150 mg once daily and 67.2–69.1% at 200 mg twice daily. Dyspareunia reduction responder rates were 45.2–45.9% at 150 mg once daily and 58.1–60.0% at 200 mg twice daily. The most common AE was hot flashes—reported among 90% of participants. The BMD in the lumbar spine was decreased from baseline: 20.63% for the lower dose recipients and 23.60% for the higher dose recipients. There were also increases from baseline in total cholesterol, LDL cholesterol, HDL cholesterol and triglycerides after the year of treatment [59].

### 5.2. Relugolix

Relugolix is another orally active, nonpeptide GnRH-receptor antagonist which was already approved in Japan in 2019 for symptoms associated with uterine fibroid treatment after the study by Osuga et al. comparing Relugolix with leuprorelin injections for uterine leiomyomas [60]. The FDA has also approved a combination of Relugolix, estradiol and norethindrone acetate for the management of heavy menstrual bleeding associated with uterine leiomyomas in premenopausal women. The approval was based on two randomized, double-blind, placebo-controlled studies: LIBERTY 1 [61] and LIBERTY 2 [62]. There are currently ongoing studies on its efficiency as a treatment for endometriosis-associated pain. Recently, on the 5th of August, USFDA approved Relugolix 40 mg, estradiol 1 mg and norethindrone acetate 0.5 mg as a one-pill, once-a-day therapy for the management of moderate to severe pain associated with endometriosis in pre-menopausal women, with a treatment duration of up to 24 months on the endometriosis indications. The effects of Relugolix on pain reduction were studied in a randomized, double-blind, placebo-controlled study by Osuga et al. Three dose levels of Relugolix were compared with a placebo and Leuprorelin to evaluate its safety and efficiency. The patients in their study included premenopausal women aged 20 to 50 years with endometriosis-associated pain (EAP) and dysmenorrhea. Patients received Relugolix daily in three oral dose levels: 10 mg (n = 103), 20 mg (n = 100) or 40 mg (n = 103); a placebo (n = 97); or Leuprorelin 3.75 mg (n = 80) as monthly subcutaneous injections for 12 weeks [63]. In another study by Osuga et al., which was the continuation of the preceding study, patients who had completed the 12 weeks of therapy continued to receive Relugolix, a placebo, or Leuprorelin in the same doses for an additional 12 weeks. There was a follow-up period of 4 weeks. Overall, the treatment duration was 24 weeks [63]. The data from both studies were combined to analyze Relugolix’s safety, efficacy and pharmacodynamics during 24 weeks of oral administration [63,64]. The pelvic pain was measured daily by VAS scores from the time of obtaining the informed consent, that is, the beginning of the study, to the end of the treatment period. The VAS scores tended to decline in time for both pelvic pain and dysmenorrhea in the Relugolix and Leuprorelin groups. Additionally, dose-dependent manner was observed. After a 12-week treatment period, the results as the changes in a VAS score were as follows: 3.8 mm in the placebo group; 6.2 (10-mg), 8.1 (20-mg) and 10.4 mm (40-mg) in the Relugolix groups; and 10.6 mm in the Leuprorelin group. In the Relugolix groups, the incidences of treatment-emergent adverse events (TEAEs) were similar to the Leuprorelin group but, more frequent in comparison to the placebo. The most frequent TEAEs (≥10%) included nasopharyngitis, headaches, menstrual abnormalities, hyperhidrosis, loss of BMD and hot flashes. Similar side effects were observed in the Leuprorelin group, with a frequency comparable to the Relugolix 40 mg group. The final conclusions of the Osuga et al. studies proved that oral administration of Relugolix appeared efficient and well-tolerated in a dose-response manner. Relugolix 40 mg in efficiency and AEs are comparable with Leuprorelin. Once-daily administered oral Relugolix was not inferior to monthly Leuprorelin injections for treating endometriosis-associated pelvic pain. After the patients had stopped taking the medication, the recurrence of menses was observed earlier in patients taking Relugolix than in those taking Leuprorelin. This result proves to be very beneficial for women who plan to conceive after treatment. Both drugs had similar safety profiles [63,64].

There are several phase 3 trials evaluating the efficacy of Relugolix in various indications completed. They include SPIRIT 1 and SPIRIT 2 [65,66]. Premenopausal women aged 18–50 years old with endometriosis diagnosed in the past 10 years, dysmenorrhea and NMPP were included in the trial. In both studies, participants were randomly divided 1:1:1 into three groups for 24 weeks of trial. Group 1 received Relugolix combination therapy (Rel-CT: Relugolix 40 mg, estradiol 1 mg, norethindrone acetate 0.5 mg) once a day, in oral administration. In group 2, Relugolix 40 mg was applied orally for 12 weeks in monotherapy, followed by Rel-CT for another 12 weeks. The last 33% of participants received a placebo. The primary endpoint was to compare Rel-CT (group 1) and the placebo on the level of dysmenorrhea and NMPP after 24 weeks, using the NRS score measured daily. The response was defined as achieving a clinically meaningful reduction from the primary level in NRS score without increased analgesic use. Group 2 (delayed Rel-CT) was included in order to compare AEs (vasomotor symptoms and loss in BMD) between Rel-CT and Relugolix monotherapy in week 12. The proportion of responders in group 2 compared to the placebo was a secondary endpoint. Main secondary outcomes included changes in the Rel-CT group vs. the placebo in the following: EHP-30 pain domain scores, average pain NRS scores and analgesics (including opioid) use. Safety endpoints included undesirable events and changes in BMD. In both trials, all the patients were randomized, and both primary endpoints (reduction in dysmenorrhea and reduction in NMPP) were achieved. In SPIRIT 1, 638 participants were included, of which 98 terminated early and 3 were not treated. This resulted in 181 patients completing treatment with Rel-CT (group 1), 182 with delayed Rel-CT (group 2) and 174 with the placebo. A reduction in dysmenorrhea criterion was met by 75% in group 1 vs. 27% in the placebo group. NMPP responses were 59% for Rel-CT therapy and 40% for the placebo. In group 2, responses for dysmenorrhea and NMPP were, respectively, 72% and 58%.

In SPIRIT 2, 623 patients were enrolled, 115 did not complete the treatment and 1 was not treated. After 24 weeks, there were 174 patients in group 1, 165 in group 2 and 168 in the placebo group. For dysmenorrhea, 75% of patients responded in the Rel-CT group, while 30% responded for the placebo. In the case of NMPP, the responses for Rel-CT amounted to 66% vs. 43% for the placebo. In group 2, the response criteria were met by 73% for dysmenorrhea and 53% for NMPP. Patients receiving Rel-CT at week 24 presented better daily functioning, assessed by the EHP-30 score; moreover, a higher number of patients were opioid-free in both SPIRIT 1 and SPIRIT 2 trials. Undesirable effects included mainly headaches, hot flashes and nasopharyngitis. Changes in BMD were overall comparable with the placebo.

Conducted trials demonstrate that one-a-day, oral Rel-CT therapy significantly improves patients’ quality of life, lowers pain levels and is generally well tolerated, causing a minimal loss in BMD. The SPIRIT extension trial, planned for 80 weeks, will provide us with additional effectiveness and safety data [65,66].

### 5.3. Linzagolix

Linzagolix is a new orally active GnRH antagonist, not yet approved by the FDA and is currently under clinical examination. It is associated with the reduction of pain and the degree of growth of endometriotic lesions [50]. The first study describing the impact of Linzagolix on EAP was conducted by Donnez et al. [67]. The study included premenopausal women from the United States of America (USA) and Europe aged between 18 and 45 years, with a confirmed surgical diagnosis of endometriosis in the previous 10 years and currently experiencing moderate to severe EAP. Patients were assigned to six treatment groups: placebo, fixed-dose (FD) Linzagolix at 50 mg, 75 mg, 100 mg, and 200 mg, and a titrated-dose (TD) group starting at 75 mg. Subjects in the placebo arm received the placebo for 12 weeks, after which they were crossed over to 100 mg Linzagolix for 12 weeks. Women in the placebo group after 12 weeks started taking 100 mg of Linzagolix for the next 12 weeks. The results after 12 weeks showed that the proportion of patients taking analgesics decreased. Before the start of the study, 95% of patients were receiving pain relief therapy until the EAP. At week 12, 91.7% of the placebo group reported taking analgesics, while the 75, 100 and 200 mg doses ranged from 68.9% to 76.1%. It is worth noting that, after 12 and 24 weeks of treatment, there was a decrease in the thickness of the endometrium and the volume of the uterus, which was most noticeable when using the drug at a dose of 100 and 200 mg. Linzagolix has been shown to be effective in relieving endometriosis-associated pain and, therefore, significantly improve the quality of life and comfort of patients.

Linzagolix was used in a follow-up, prospective, randomized, double-blind study in subjects who completed the 6-month treatment period in study 18-OBE2109-002-Edelweiss 2 [68]. Patients after 6 months of participation in the study were able to decide either to end the treatment and proceed to the follow-up process or to take part in an additional study and extend the treatment by 6 months. They were taking Linzagolix 75 mg alone (with ABT placebo) or 200 mg in combination with ABT once daily for 6 months. Patients assessed these complaints using the Verbal Rating Scale (VRS) ranging from 0 to 3 using electronic diaries. The following parameters were also assessed: dysmenorrhea, NMPP, dyschezia, overall pelvic pain, interference of pain with the ability to perform daily activities, dyspareunia, no analgesic use for endometriosis-associated pain and no opiate use for endometriosis-associated pain. The study results, which were published by the research sponsor ObsEva, showed that pelvic pain in subjects taking 75 mg of Linzagolix decreased after 12 weeks in 61.51% of patients, and after 24 weeks, improvement was observed in 77.3% of patients [68]. At the dose of 200 mg, pain was reduced in 56.3% of patients after 12 weeks and in 77.3% after 24 weeks of therapy. As well, 68.2% of the patients reported an improvement in dysmenorrheal pain with the 75 mg dose, and 58.2% after 24 weeks. When taking the 200 mg dose, 78.9% of respondents improved after 12 weeks, and 84.1% after 24 weeks.

Currently, further investigation is being conducted into the efficacy and safety of both Linzagolix ad-ministered orally once daily for 3 months and Linzagolix in the dose of 75 mg alone or of 200 mg in combination with ABT (ABT: estradiol (E2) 1 mg/norethisterone acetate (NETA) 0.5 mg) versus a placebo in the management of moderate to severe EAP [69]. The two most important measures that will be compared throughout the whole study period, that is, from the baseline to the end of the third month of treatment, are dysmenorrhea and NMPP. Additionally, dysmenorrhea, NMPP, dyschezia, overall pelvic pain, interference of pain with the ability to perform daily activities, dyspareunia, no analgesic use for endometriosis-associated pain and no opiate use for endometriosis-associated pain from baseline to month 6 will also be assessed. The planned end of the study was scheduled for July 2022; therefore, a prompt publication of the results is expected. The study included women aged 18 to 49 who had regular menstrual cycles, body mass index (BMI) ≥ 18 kg/m^2^ at the screening visit and who suffered from moderate to severe endometriosis-related pain during the screening period. The results of the study are pending. In March 2022, the sponsor of the ObsEva study announced the results for Linzagolix 200 mg with ABT and Linzagolix 75 mg without ABT in the phase 3 EDELWEISS 3 trial in patients with moderate to severe endometriosis-associated pain [70]. There was an improvement in the two most important points assessed in the study: the reduction of dysmenorrhea and the reduction of NMPP, compared to placebo, which were observed for both doses after 1 and 2 months of treatment. The improvement was also noticed in the case of dyschezia and worse pelvic pain. The quality of life also improved as the pain associated with endometriosis was reduced. What is more, after 6 months of treatment, the initial number of intended surgical procedures for endometriosis also decreased. The described research creates the possibility of controlling the pain associated with endometriosis progressing over time.

At the end of 2021, the results of the study on Linzagolix in the treatment of uterine adenomyosis were presented [71]. The research group consisted of only eight women aged 37 to 45. All of the patients were pre-menopausal. The study participants suffered from uterine adenomyosis on MRI. Each of the patients struggled with heavy menstrual bleeding and pelvic pain. In the study, women received 200 mg of the drug once a day for 12 weeks and then the dosage was reduced to 100 mg once a day for the next 12 weeks. After 24 weeks from the beginning of the study, the average pelvic volume of women decreased significantly (333 ± 250 cm^3^ at the start of the study and 204 ± 126 cm^3^ at 24 weeks). Such symptoms as pelvic pain, dyspareunia and dyschezia also decreased. At week 24, the mean global pelvic pain scores were 0.6 ± 0.7, whereas in the beginning, the scores were 8.4 ± 1.1. The mean dyspareunia scores were 0.1 ± 0.2 at 24 weeks, whereas at the baseline, the results were 1.2 ± 1.2. The mean dyschezia scores were 1.6 ± 2.2 compared to 2.7 ± 2.8 in the beginning. The above study clearly indicated that Linzagolix significantly reduced patients’ complaints, which may suggest its effectiveness in the treatment of adenomyosis. Moreover, the obtained results may also suggest that the drug will be effective in the course of treatment for endometriosis. Importantly, both adenomyosis and endometriosis were treated as two separate entities. In adenomyosis, the endometrium (endometrial tissue) is implanted deep into the myometrium. In endometriosis proper, the mucosal cells (endometrium) are found outside the uterus. Thus, the promising results of clinical trials in the case of adenomyosis, the specific form of endometriosis, require further analysis, as they may prove to be a breakthrough in the treatment of subsequent gynecological diseases.

Jacques Donnez et al. in 2022 conducted a study in which Linzagolix with and without hormonal ABT was implemented to treat symptomatic uterine fibroids. The treatment with PRIMROSE 1 and PRIMROSE 2 lasted 52 weeks. These were two identical studies, randomized, parallel, double-blind, placebo-controlled, phase 3 trials. They included women aged 18 and older with US-confirmed fibroids, who also suffered from heavy menstrual bleeding assessed by the alkaline hematin method bringing 80 mL or more of menstrual blood per cycle for at least two cycles. Participants in the study were required to have at least one myoma with a diameter not smaller than 2 cm (or multiple small fibroids with a calculated uterine volume greater than 200 cm^3^) and no myoma greater than 12 cm in diameter. The subjects were recruited from May 2017 to October 2020. Ultimately, 511 women were enrolled in the PRIMROSE 1 study and 501 women were enrolled in the PRIMROSE 2 study. There was a reduction in menstrual bleeding in all groups treated with Linzagolix and Linzagolix alone with and without ABT compared to the placebo (all *p* ≤ 0.003). After 24 weeks, the group receiving 200 mg of Linzagolix demonstrated a decrease in the mean uterine volume by 31% in the PRIMROSE 1 treatment and 43% in the PRIMROSE 2 treatment. Furthermore, a reduction in the mean uterine volume (15%) was also observed in the treatment alone in the 100 mg and 200 mg doses with the addition (with once-per-day hormonal ABT). Therefore, clearly, Linzagolix (100 mg or 200 mg) with or without adjuvant therapy offers opportunities to significantly improve the quality of life of women with symptomatic uterine fibroids [72]. All conducted studies prove the usefulness of Linzagolix in the course of treatment of gynecological diseases and in the treatment of endometriosis in particular.

Comparison of GnRH antagonist trials presented in Table 2 [53,54,55,63,64,65,66,67,68,69,70,71,72]. The list includes the location of the study, age, size of the group and the main effects after treatment.

## 6. Discussion

The main purpose of the present review was to indicate the efficacy of GnRH antagonists in the course of treatment of pain associated with endometriosis. The traditional treatment of endometriosis includes drugs and surgical management. Laparoscopy was recommended for patients who failed pharmacological treatment, yet regrettably, the surgery did not completely reduce pain associated with the illness. While pharmacological management is still the first-line approach to the treatment of endometriosis-related pain, surgery should be reserved for initial diagnosis and situations where the first-line treatment has failed. Additionally, despite surgical intervention, pain reoccurs in 20% to 56% of the women in the following 5 years, and with a high plausibility of the development of postoperative adhesions and the necessity for an additional surgery affects nearly two-thirds of the women [73]. Nevertheless, due to the fact that the available pharmacological therapies bring side effects, there is a need for the continued search for new options [27]. All recognized methods of pharmacological treatment seem to have very similar therapeutic effects and that is why the use of GnRH antagonists in the therapy of endometriosis has grown in importance lately. What is more, since the introduction of GnRH antagonists, new options for women with endometriosis have been created, especially for those with progestin resistance or progestin-related side effects [74]. There are two oral GnRH antagonists available: Elagolix [75] and Relugolix [76], already approved by the USFDA. The third—Linzagolix—is currently under examination [66] after the successful third-phase trial. Currently, two out of three known GnRH antagonists have defined doses: Elagolix, 150 mg recommended once daily for ≤24 months in patients who also suffer from dyspareunia; Elagolix, at a higher dose, 200 mg recommended twice daily for ≤6 months. In the case of Relugolix, the following doses and times are recommended: Relugolix (40 mg, estradiol 1 mg, and norethindrone acetate 0.5 mg) as a one-pill, once-a-day therapy for the management of moderate to severe pain associated with endometriosis in pre-menopausal women, with a treatment duration of up to 24 months. The dosage of Linzagolix is still undefined.

GnRH antagonists may be beneficial in many ways when compared to other available older drugs. The use of GnRH antagonists appears to reduce the doses of administered analgesics. In the studies of Taylor, H.S. et al., EM-I and EM-II indicated that women who took the higher doses of Elagolix needed a significantly lower amount of pill counts of NSAIDs, opioids or both combined when compared to the placebo recipients. Importantly, women from the lower-dose group did not significantly reduce their use of analgesics. Additionally, patients who took 200 mg of Elagolix were able to observe significant reductions in the dyspareunia score just after halfway through the trial. Moreover, Elagolix showed meaningful reduction in dysmenorrhea and NMPP. Clinical trials demonstrated 46.4–75.8% (dose-dependent) reduction in dysmenorrhea and about 50% improvement in NMPP compared to women who received placebo. What is more, when still under examination in phase 2 studies, Elagolix, unlike progestins [77], did not cause irregular bleeding from uterine or general increase in bleeding. Overall, Elagolix recipients experienced a reduction in bleeding with fewer days of moderate to heavy bleeding. Most patients who took Elagolix had regular but prolonged menstrual cycles [53].

GnRH analogs: agonists and antagonists are a second-line therapy in the course of endometriosis therapy [78]. They work by down-regulating the hypothalamic–pituitary–gonadal axis, suppressing ovulation and reducing estrogen levels, and due to their different mechanisms of action, they are frequently being compared. Elagolix has two main advantages in comparison with conventional GnRH agonists. Firstly, the drug is administered orally while GnRH agonists are mostly used as long-acting depot formulations, subcutaneous implants or daily nasal solutions, which are less convenient for patients. Secondly, Elagolix has a short half-life (~6 h) which allows a more rapid elimination from the system. If the treatment needs to be discontinued for any reason [53], Elagolix does not block the pituitary axis completely, and a remaining level of estrogen is able to fulfill its protective function, unlike the case of the treatment with GnRH agonists [53]. In the phase 3 trial (NCT02655237), Relugolix (40 mg once daily) achieved a similar proportion of heavy menstrual bleeding in responders at 6–12 weeks compared to Leuprorelin administered by monthly injection (82% versus 83%). Moreover, this GnRH antagonist had a faster action onset than Leuprorelin. Additionally, E2 levels and menses resumed more quickly after the discontinuation of Relugolix when compared to Leuprorelin discontinuation [79].

Elagolix, unlike other GnRH antagonists, is prescribed as monotherapy without hormone replacement therapy. Furthermore, Relugolix as a once-a-day self-administered oral medication possesses an excellent adherence profile. In addition, the principles of the Relugolix therapy are more comprehensible for patients and, thus, they are in compliance with the medical recommendations, which is an improvement over Elagolix. Moreover, Elagolix has a more complex treatment regimen in comparison to Relugolix and may require a dose adjustment depending on the patient’s condition. Due to the positive results in studies and the minimal changes in BMD, which are one of the most serious side effects of GnRH antagonists, Linzagolix in the dose of 200 mg with ABT may have the potential to replace Elagolix or Relugolix. It is suggested that those doses of Linzagolix might be used for long-term treatment, unlike other GnRH antagonists. Moreover, the greatest advantage of Elagolix therapy is the fact that it is the longest-known drug and is safely used in the treatment of endometriosis.

The treatment with GnRH antagonists was also connected with many side effects. During Elagolix trials, changes in the lipid profile, such as increased LDL cholesterol and total cholesterol levels, were observed [56]. Some trials indicate a higher risk of coronary heart disease among women with endometriosis, but this observation still requires further research into the changes in lipid levels and their influence on long-period cardiovascular risk [80].

Moreover, the side effects of GnRH antagonists include the reduction of BMD and the risk of osteopenia and osteoporosis as a result of hypoestrogenemia. Therefore, Relugolix treatment is currently limited to 6 months [64], and Elagolix, depending on doses: 150 mg once daily up to 24 months and twice daily 200 mg up to 6 months. As a result of the data collected from the EDELWEISS 3 trial, Linzagolix 200 mg with ABT may be used as a long-term treatment because of its small impact on BMD [70].

What is more, only about 1% of the women who attended EM-I, EM-II, EM-III and EM-IV trials, and took 150 mg, and less than 3% of those taking 250 mg of Elagolix, needed to discontinue the study because of the incidence of hot flashes [56]. During Linzagolix therapy, hot flashes were reported with a frequency of 42.1% in women taking the 200 mg dose and 19.3% in women taking the 75 mg dose [67]. In addition, headaches were also reported as one of the most common side effects. TEAEs were reported in each study week, but none of them were associated with the treatment proper.

We should not forget about the limitations of conducted studies. In the Daisy Petal trial, the main limitation was the fact that the participants were recruited from patients who previously took part in the Elagolix trial [53]. The Tulip PETAL trial enrolled mostly patients from Eastern Europe, and the outcome may differ among the USA or Western Europe population [56]. For example, women from Central Eastern Europe had generally lower BMI than American women [57]. EM-II, EM-III and EM-IV were limited by the entry criteria—only premenopausal women aged from 18 to 49 with surgically confirmed lesions were included in the studies. In addition, the time of intervention in the studies varied. Elagolix treatment was also not conducted among women with a Z score of less than −1.5 for BMD or in women with large endometriomas [56,59]. The studies by Yutaka Osuga et al. also have some limitations. The results should be confirmed in the future because the trials included women of only Japanese ethnicity. What is more, approximately 40% of subjects were diagnosed based on clinical signs and symptoms alone, because endometriosis is rarely diagnosed by laparoscopy or laparotomy at the beginning of treatment.

Another issue associated with GnRH antagonist therapy is their cost. For example, Elagolix, produced by AbbVie Inc. in the North Chicago, IL, USA, is priced at about USD 10,000 per year (USD 2500 per 3 months), which can be unaffordable for patients or cause a budget burden for tax-funded healthcare systems, especially in low-income countries [81]. Sze Wan Hung et al. analyzed the approximate costs of 3-month endometriosis therapy with different agents. According to their study, first-line treatment, which is oral estrogen + progestin therapy, is less than USD 100, while progestin therapy, depending on the administration, varies from USD 100– USD499 for oral or intramuscular administration to $500–$1999 for intrauterine systems. GnRH agonist goserelin (subcutaneous injections) and nafarelin acetate (nasal spray) prices are USD 100–USD 499 and >USD 5000, respectively. According to that, nafarelin acetate therapy is one of the most expensive of all, oscillating in the same price range as LHRH agonist leuprolide in intramuscular injections. Other endometriosis therapies (Danazol (oral administration) and a combination of Leuprolide and Norethindrone (intramuscular injections)) seem to be in the same price range as Elagolix, which is USD 2000–USD4999 per three months. This makes GnRH antagonists not the most expensive group; however, much more expensive than current first-line endometriosis treatment [82].

Currently, there is a clinical study conducted to evaluate the safety and efficacy of Elagolix in combination with concomitant hormonal ABT among premenopausal participants with moderate to severe endometriosis-associated pain. The trial is expected to terminate in 2023 [83]. In the Elaris Uterine Fibroids 1 and 2 (UF-1 and UF-2) trials, one of the assessed aspects was the hypoestrogenic effects in women who received Elagolix with ABT, as compared with Elagolix alone. The decreases from baseline in BMD that occurred in women who received Elagolix alone were significantly greater than in those who received Elagolix with ABT and also significantly greater than in those who received a placebo [84].

What is more, there is a currently undergoing study assessing the contraceptive efficacy of Relugolix combination therapy (Relugolix 40 mg, estradiol 1 mg and norethindrone acetate 0.5 mg administered orally daily for one year) by the At-Risk Pearl Index (PI) in women with uterine fibroids or endometriosis and who are at risk for pregnancy [65,85]. This study may demonstrate other therapeutic benefits of Relugolix among women of reproductive age. Additionally, other drugs are undergoing examination for endometriosis treatment. One of them is selective estrogen receptor modulators (bazedoxifene, raloxifene) and progesterone receptor modulators (mifepristone, ulipristal). Raloxifene did not seem effective despite the promising animal models, as in women with postoperative raloxifene therapy the endometriosis-associated pain returned sooner than the placebo. Bazedoxifene has the potential to become a new treatment option; however, it still requires randomized clinical trials. Considering progesterone receptor modulators, mifepristone is the most popular one to examine. Ulipristal appears to be effective; however, it caused endometrial thickening, which resulted in the discontinuation of the study [86].

## 7. Conclusions

In conclusion, pain in endometriosis is one of the most challenging problems; however, the patients still cannot be offered a satisfactory treatment. GnRH antagonists appear to be a promising group of drugs but they also demonstrate numerous side effects, mostly associated with hypoestrogenic states, such as decreased BMD, hot flashes and mood or sleep disturbances. However, their pharmacokinetic profile tends to be less damaging in comparison to GnRH agonists. Based on the knowledge concerning the possible side effects, patients individually should decide whether to choose or not to choose this kind of pharmacological therapy among others. The main advantages of GnRH antagonists observed so far include the reduction of dysmenorrhea and NMPP, but further studies are necessary to compare the efficacy and safety of different analogs of GnRH antagonists.

## Figures and Tables

**Table 1:** Mechanism of action of drugs used in endometriosis treatment.

Drug Category	Mechanism of Action
Non-steroidal anti-inflammatory drugs	Reversibly inhibit COX-1 and COX-2, which results in a decreased level of prostaglandin formation
Combined estrogen–progestin contraceptives	Inhibit FSH and LH, decrease cell proliferation and enhance endometrial apoptosis
Progestin-only preparations	Inhibit FSH and LH and stimulate atrophy or regression of endometrial lesions
Androgens	Antiestrogens inhibit enzymes involved in steroid formation and decrease the release of gonadotropin
Aromatase inhibitors	Block conversion of androgenesis to estrogen, which decreases endometrial proliferation
GnRH agonists	Chronic administration inhibits steroidogenesis due to the reduced LH and FSh levels. The initial hormone flare is characteristic of GnRH agonists

**Table 2:** The comparison of GnRH antagonists—randomized clinical trials.

The Name of the Drug	The Name of the Randomized Clinical Trial and the Phase	The Location	The Population	The Sample Size	The Results
Elagolix	phase 2 Daisy PETAL trial	USA	women between 18 and 49 years old	252 participants (169 completed trial)	reduction in dysmenorrhea, NMPP and dyspareunia, EAP
Elagolix	phase 2 Lilac PETAL trial	USA	women between 18 and 49 years old	155 participants (127 completed trial)	reduction in dysmenorrhea and NMPP
Elagolix	phase 2 Tulip PETAL trial	USA	women between 18 and 45 years old	174 participants (164 completed trial)	reduction in monthly mean pelvic pain and EAP
Elagolix	phase 3 Elaris Endometriosis 1 trial (EM-I)	USA, Canada	women between 18 and 49 years old	872 participants (653 completed trial)	reduction in dysmenorrhea and NMPP
Elagolix	phase 3 Elaris Endometriosis 2 trial (EM-II)	Argentina, Austria, Australia, Brazil, Czech Republic, Hungary, Italy, New Zealand, Poland, South Africa, Spain, USA, the United Kingdom	women between 18 and 49 years old	815 participants (632 completed trial)	reduction in dysmenorrhea and NMPP
Elagolix	phase 3 Elaris Endometriosis 3 trial (EM-III)	USA, Puerto Rico, Canada.	women between 18 and 49 years old	287 participants (226 completed trial)	reduction in dysmenorrhea, NMPP and dyspareunia
Elagolix	phase 3 Elaris Endometriosis 4 trial (EM-IV)	North and South America, Europe, Australia/New Zealand, South Africa	women between 18 and 49 years old	282 participants (232 completed trial)	reduction in dysmenorrhea, NMPP and dyspareunia
Relugolix	phase 3 trial	Japan	women aged 20 and older	281	reduction in EAP
Relugolix	phase 3 Liberty 1 trial	USA, Brazil, Italy, Poland, South Africa, the United Kingdom	women between 18 and 50 years old	388 participants (308 completed trial)	decreased menstrual bleeding and preserved BMD in women with uterine fibroids
Relugolix	phase 3 Liberty 2 trial	USA, Belgium, Brazil, Chile, Czech Republic, Hungary, Poland, South Africa	women between 18 and 50 years old	382 participants (302 completed trial)	decreased menstrual bleeding and preserved BMD in women with uterine fibroids
Relugolix	phase 2 trial	Japan	women between 20 and 50 years old	487 participants	reduction in EAP
Relugolix	phase 3 SPIRIT 1 trial	USA, Canada, Bulgaria, Polan Ukraine, Czech Republic, Finland, Hungary, Portugal, Spain, South Africa	women between 18 and 50 years old	638 participants (540 completed trial)	reduction in dysmenorrhea, NMPP and EAP
Relugolix	phase 3 SPIRIT 2 trial	Australia, Brazil, Chile, Czechia, Georgia, Italy, New Zealand, Poland, Romania, Sweden, USA	women between 18 and 50 years old	623 participants (508 completed trial)	reduction in dysmenorrhea, NMPP and EAP
Linzagolix	phase 2b EDELWEISS 1 trial	USA, Europe	women between 18 and 45 years old	328 participants (323 completed trial)	reduction in EAP and improved quality of life
Linzagolix	phase 3 EDELWEISS 2 trial	USA, Canada, Puerto Rico	women between 18 and 50 years old	30 participants	reduction in pelvic pain, dysmenorrheal pain
Linzagolix	phase 3 EDELWEISS 3 trial	USA, Poland, Czech Republic, Austria, Romania, Ukraine, France, Spain, Bulgaria	women between 18 and 50 years old	288 participants	reduction in dysmenorrhea and NMPP, improvement in dyschezia and lowered worst pelvic pain
Linzagolix	pilot study in the single center	Europe	women between 37 and 45 years old	10 participants (8 completed trial)	average pelvic volume of women decreased, reduced pelvic pain, dyspareunia, and dyschezia
Linzagolix	phase 3 PRIMROSE 1 trial	USA	women aged 18 and older	574 participants (511 completed trial)	decrease in the mean uterine volume by 31%
Linzagolix	phase 3PRIMROSE 2 trial	USA, Europe	women aged 18 and older	534 participants (501 completed trial)	decrease in the mean uterine volume by 43%

## Data Availability

Not applicable.

## Supplementary Materials

The following supporting information can be downloaded at: https://www.mdpi.com/article/10.3390/jcm12031008/s1, Table S1: Terms and Boolean operators used in the methodology.
