# Supplementary material for: Gonadotropin-Releasing Hormone Antagonists—A New Hope in Endometriosis Treatment?
Source: J Clin Med. 2023 Jan 28;12(3):1008. doi: 10.3390/jcm12031008 (PMC9918258; doi:10.3390/jcm12031008)
Supplement: Supplementary file 1 [file jcm-12-01008-s001.zip › jcm-2136381-supplementary.pdf]

Journal of
Clinical Medicine

Materials and methods

The studies cited in the presented review were selected from PUBMED, Google Scholar and Science Direct databases. Also, the websites including https://clinicaltrials.gov/ and https://www.cochranelibrary.com/central/about-central were checked in details. Terms used by us were created by combining all words connected with GnRH antagonists, which we were describing in our manuscript, by using boolean operator “OR”. Among these terms were for example: “GnRH antagonists”, "relugolix", "elagoilix". Thereafter we were adding new words of interest with the use of the boolean operator "AND". These words were changing depending on the part of the manuscript, we were working on. Among them were for example: “endometriosis”, “endometriosis treatment”, and more specific including: “relugolix” or “elagolix” and others, which are connected to selected sections. All terms and Boolean operators used with them were shown in the table S1.

Table S1. Terms and Boolean operators used in the methodology.

OR	AND
GnRH analogs	endometriosis
GnRH antagonists	endometriosis treatment
GnRH agonists	pelvic pain
elagolix	dysmenorrhea
relugolix	endometriosis-associated pain
linzagolix	pain associated endometriosis
-	chronic pelvic pain

Even if the paper was designed as narrative review, we applied the rules of paper selection listed below.

Inclusion criteria:

- The types of included studies: clinical trials, retrospective studies, reviews;
- More than one patient described in the study;
- No limitations of the year of published study were used

Exclusion criteria:

- Articles not written in English
- Conference abstracts only
- Study cases
- Duplicated papers
